# A novel modified-indirect ELISA based on spherical body protein 4 for detecting antibody during acute and long-term infections with diverse *Babesia bovis* strains

**DOI:** 10.1186/s13071-017-2016-9

**Published:** 2017-02-13

**Authors:** Chungwon J. Chung, Carlos E. Suarez, Carey L. Bandaranayaka-Mudiyanselage, Chandima-Bandara Bandaranayaka-Mudiyanselage, Joanna Rzepka, TJ Heiniger, Grace Chung, Stephen S. Lee, Ethan Adams, Grace Yun, Susan J. Waldron

**Affiliations:** 1 Washington State University, Pullman, WA USA; 2 VMRD, Inc., Pullman, WA USA; 3 USDA-ADRU, Pullman, WA USA; 4 University of Idaho, Moscow, ID USA; 5 Tick Fever Center, Wacol, Australia

**Keywords:** *Babesia bovis*, Modified indirect enzyme-linked immunosorbent assay, Diagnosis, Spherical body protein-4

## Abstract

**Background:**

Cattle persistently infected with *Babesia bovis* are reservoirs for intra- and inter-herd transmission. Since *B. bovis* is considered a persistent infection, developing a reliable, high-throughput assay that detects antibody during all stages of the infection could be pivotal for establishing better control protocols.

**Methods:**

A modified indirect enzyme-linked immunosorbent assay (MI-ELISA) was developed using the spherical body protein-4 (SBP4) of *B. bovis* to detect antibody against diverse strains through all infection stages in cattle. This SBP4 MI-ELISA was evaluated for sensitivity and specificity against field sera from regions with endemic and non-endemic *B. bovis*. Sera were also evaluated from cattle infected experimentally with various doses and strains during acute and persistent infection with parasitemia defined by nested PCR.

**Results:**

The format variables for SBP4 MI-ELISA were optimized and the cutoff for positive and negative interpretation was determined based on receiver operating characteristic curve analysis using *B. bovis* positive and negative sera tested in the reference immunofluorescence assay (IFA). The diagnostic specificity of the SBP4 MI-ELISA using IFA-negative sera collected from Texas was 100%, significantly higher than the cELISA (90.4%) based on an epitope in the rhoptry-associated protein-1 (RAP-1 cELISA). The diagnostic sensitivity of the SBP4 MI-ELISA was 98.7% using the IFA-positive sera collected from several areas of Mexico, in contrast to that of the RAP-1 cELISA at 60% using these same sera. In cattle infected with low and high doses of three *B. bovis* strains, the SBP4 MI-ELISA remained antibody positive for 11 months or more after initial detection at 10 to 13 days post-inoculation. However, the RAP-1 cELISA did not reliably detect antibody after eight months post-inoculation despite the fact that parasitemia was occasionally detectable by PCR. Furthermore, initial antibody detection by RAP-1 cELISA in low-dose infected animals was delayed approximately nine and a half days compared to the SBP4 MI-ELISA.

**Conclusions:**

These results demonstrate excellent diagnostic sensitivity and specificity of the novel SBP4 MI-ELISA for cattle with acute and long-term carrier infections. It is posited that use of this assay in countries that have *B. bovis*-endemic herds may be pivotal in preventing the spread of this disease to non-endemic herds.

**Electronic supplementary material:**

The online version of this article (doi:10.1186/s13071-017-2016-9) contains supplementary material, which is available to authorized users.

## Background

Bovine babesiosis, caused by protozoa of the genus *Babesia* (order Piroplasmida, phylum Apicomplexa), is an economically important tick-borne disease of cattle, particularly in tropical and subtropical areas of the world [1, 2]. Of the various *Babesia* species, *B. bovis* and *B. bigemina* are widely distributed and of major importance in Africa, Asia, Australia, and Central and South America [2]. The prevalence of *B. bovis* and *B. bigemina* in various countries is closely associated with the presence of the tick *Rhipicephalus microplus*, which is the main vector of *Babesia* transmission to cattle and related species [3]. *Babesia bovis* is generally more pathogenic than other bovine *Babesia* species [2], with infection characterized by high fever, ataxia, anorexia, circulatory shock, and occasionally central nervous system signs due to sequestration of parasitized erythrocytes in cerebral capillaries [1, 2]. *Babesia* species that cause severe disease in naïve cattle are a potential threat to *Babesia*-free or non-endemic areas of the world that have competent tick vectors, including the United States.

The long-term persistence of *B. bovis* in cattle infected with a moderately attenuated strain or vaccinated with a modified live vaccine (MLV) strain has been demonstrated [4, 5]. Calves infected with Mo7 strain, attenuated by cloning and in vitro passage, remain infected for longer than ten months based on PCR and other evidence [5]. Friesian cattle vaccinated with an attenuated strain had evidence of parasitemia for up to 47 months as determined by sub-inoculation into splenectomized calves [4]. These two experiments, though limited, provide strong evidence for long-term persistence of *B. bovis* infection in cattle. In light of this, an ideal serological assay should target a *B. bovis* antigen that is stably expressed in infected animals by multiple parasite stages for an extended period, and be able to detect specific antibodies throughout all stages of infection. However to our knowledge, there is no report on long-term (>100 days post-infection or post-vaccination) monitoring of *B. bovis-*specific antibody responses in infected cattle. One study monitored *B. bovis-*specific antibody responses through 98 days after challenge with a virulent strain, T2Bo, using the cELISA based on the rhoptry-associated protein-1 (RAP-1) [6]. Another study monitored antibody responses based on the reference immunofluorescence assay (IFA) through 60 days post-vaccination [7]. In these two studies, both the cELISA and IFA were reliable for short-term monitoring of *B. bovis*-specific antibody responses; however, their reliability for monitoring longer post-infection periods has not been determined. Further defining long-term persistence of *B. bovis* infection and longevity of *B. bovis*-specific antibody responses in cattle after infection with various attenuated vaccine strains and pathogenic strains is crucial for developing better control measures.

Several assays to detect *B. bovis*-specific antibodies have been described. Of these, the IFA is the most widely used, but low throughput and subjectivity in scoring results as positive or negative are major disadvantages to its general use for serological diagnosis of *B. bovis* infection [2]. The complement fixation (CF) test has been used in some countries to qualify animals for importation as well as for general diagnosis [2]. However, the CF test is labor-intensive, time-consuming to perform, and suffers from poor reproducibility between laboratories, perhaps attributable to inadequate standardization of both test reagents and procedure as reported in other diseases with intraerythocytic parasites [8]. Further, the CF test as formatted using guinea pig complement does not detect all bovine IgG antibody isotypes [9], possibly contributing to poor diagnostic sensitivity. Due to these disadvantages, IFA and CF tests have largely been replaced by ELISAs as the serodiagnostic tests of choice for *B. bovis* [2, 6, 10–15]. Early ELISA formats for *B. bovis* included indirect ELISAs using whole merozoite antigen [13] or recombinant subunit proteins [10, 11]. More recently, a competitive blocking ELISA (cELISA) based on a monoclonal antibody to an epitope of RAP-1 has been developed and evaluated with limited sera [6, 12]. Presently, there is no ELISA or other high throughput assay that has been systematically validated against diverse sera from cattle in different infection stages from various geographical areas [2]. ELISAs using whole parasite antigen results in relatively lower diagnostic specificity because of cross-reactivity with closely-related *Babesia* species and possibly other genera [8, 16]. If sufficiently-conserved subunit proteins containing multiple B cell epitopes are not utilized, ELISAs using subunit proteins of *B. bovis* tend to have the opposite problem of relatively lower diagnostic sensitivity due to antigenic variation among *B. bovis* strains [15]. To overcome the challenges in serodiagnosis of *B. bovis* and to improve control of infection globally, a new high-throughput assay demonstrating excellent diagnostic sensitivity and excellent *B. bovis* species specificity is needed. Such a serodiagnostic assay would detect antibody throughout all stages of infection with the vast majority (ideally all) of global *B. bovis* isolates and would not detect antibody induced by other closely related *Babesia* species.

A recent comparative study with five subunit proteins of *B. bovis* in an indirect ELISA format reported that spherical body protein-4 (SBP4) was relatively better than MSA-2c, RAP-1/CT, TRAP-T and SBP1 in both diagnostic sensitivity and specificity [15]. Further study defined that SBP4 was produced and secreted in a stable and dominant manner through all stages of the *B. bovis* life-cycle [17]. However, only 85% diagnostic concordance (84.5% sensitivity and 86.2% specificity) was observed between an SBP4-based indirect ELISA and IFA with 469 sera collected from five countries [15] suggesting the need for further improvement. The present study had three main objectives: (i) develop and evaluate a novel SBP4 MI-ELISA format using recombinant SBP4 as the coating antigen and detection conjugate to produce higher diagnostic performance than previously reported antibody assays, (ii) compare the performance of this novel SBP4 MI-ELISA to the reference IFA and to the previously developed RAP-1 cELISA using cattle sera from non-endemic, endemic and epidemic areas, and (iii) demonstrate that the novel SBP4 MI-ELISA would detect antibody in sera from cattle that had long-term infections documented by at least occasional PCR positive tests and then compare these results with those from IFA and RAP-1 cELISA.

## Methods

### DNA extraction and PCR

DNA was extracted with purification reagent from FTA cards spotted with whole blood according to the manufacturer’s instructions (FTA card and DNA purification reagent, GE Healthcare, Piscataway, NJ, USA). Briefly, blood samples were dotted on FTA cards and treated with the purification reagent to lyse erythrocytes and remove cellular proteins. Sample DNA was eluted in PCR buffer and nested PCR was performed to amplify a 291 bp fragment from the *B. bovis* RAP-1 gene according to previously reported methods [5].

### Cloning and expression of SBP4

A fusion protein that included glutathione S-transferase (GST) and the SBP4 protein of *B. bovis* (T2Bo strain) with the signal sequence deleted (GenBank accession number: XM_001610418) was cloned into the pGEX-2T vector (New England BioLabs, Ipswich, MA, USA). This recombinant (r) GST-SBP4-containing vector was transformed into BL21 cells (New England BioLabs) and expressed as follows. Briefly, 200 ml of an overnight culture of pGEX-2T-SBP4-transformed BL21 cells were inoculated into 1.8 l of LB medium (Becton, Dickinson and Company, Sparks, MD, USA) containing 0.01% ampicillin and grown at 37 °C for 3 h. Following addition of 0.048 g of isopropyl-β-D-thiogalactopyranoside (IPTG) (USBiologicals, Salem, MA, USA), the bacteria were incubated at 37 °C for an additional 5 h and then harvested by centrifugation at 4.5 × 10^3^ *g* for 25 min. The pellet was resuspended in phosphate buffered saline, sonicated on ice, and Triton X-100 (Sigma-Aldrich, St. Louis, MO, USA) was added to a final concentration of 1%. The suspension was then centrifuged at 5.7 × 10^4^ *g* for 30 min. Finally, the clarified supernatant containing the rGST-SBP4 fusion protein was collected and stored at -80 °C until used for antigen coating or further purification to make the horseradish peroxidase (HRP)-conjugated recombinant SBP-4.

### Purification of rGST-SBP4 fusion protein and conjugation with HRP

The rGST-SBP4 fusion protein was purified from the supernatant described above using glutathione-agarose beads (Sigma-Aldrich), according to the manufacturer’s instruction. Briefly, glutathione-agarose beads equilibrated with PBS containing 1% Triton-X 100 were resuspended in the supernatant containing rGST-SBP4 and agitated gently for 60 min at room temperature. The beads were pelleted by centrifugation and washed with PBS three times before the rGST-SBP4 was eluted from the beads using elution buffer containing 30 mM reduced glutathione in 50 mM Tris–HCl (pH 9.0). The eluted rGST-SBP4 fusion protein was conjugated with HRP as previously described [18], stabilized by adding heat-inactivated goat serum to a final concentration of 10%, and stored at 2–7 °C.

### Bovine sera used for assay evaluations

Negative sera were collected from 302 uninfected cattle in northwestern US herds that were maintained in barns free of *B. bovis* transmitting ticks and that had no history of clinical signs of babesiosis. Ninety-four sera were collected from Texas herds with unknown *B. bovis* status, 93 of these sera were negative by IFA and one was weakly positive by IFA. Thirty-two sera negative by *B.bovis* IFA were selected as additional negative samples from *B. bovis* endemic areas of Mexico. These 427 negative sera were used to evaluate the diagnostic specificity of the RAP-1 cELISA and the newly developed SBP4 MI-ELISA. *B. bovis*-positive sera (*n* = 826) were obtained from cattle with positive results by IFA, and were used to evaluate the diagnostic sensitivity of the SBP4 MI-ELISA and the RAP-1 cELISA. Two hundred twenty-eight of these 826 sera were from herds in Australia vaccinated with the attenuated *B. bovis* Dixie strain (*n* = 180) or challenged with the virulent W strain (*n* = 48), and the remaining 598 sera were collected from several areas in Mexico. Another 402 IFA-positive sera were selected from sequential serum collections from six experimentally infected cattle and used to further evaluate the relative sensitivity of the SBP4 MI-ELISA and the RAP-1 cELISA. Excluding the 32 negative samples at early post-infection days and 402 positive samples from experimentally infected animals, a total of 1,253 field serum samples were used to evaluate likely diagnostic sensitivity and specificity resulting in a statistical power of 95% confidence within a 2% margin of error. Twenty-five sera poaitive by *B. bigemina* RAP-1 antibody cELISA (VMRD Inc.) and 25 sera positive by *Anaplasma* MSP5 antibody cELISA (VMRD Inc., Pullman, WA, USA) were additionally included for evaluating the analytical specificity of the *B. bovis* MI-ELISA.

### Infection of calves with different *Babesia bovis* strains

Two calves were infected with the Mo7 strain [19] and two calves with the Tf-137-4 strain of *B. bovis* [20, 21] in a previous study [5]. These four calves received a low dose (5 × 10^3^) of infected erythrocytes intravenously and were monitored for signs of acute babesiosis, including parasitemia, fever, and low packed cell volume (PCV) on a daily basis for the first twenty days post-infection (DPI) and on a biweekly basis thereafter until 11 months post-infection. Two other calves were infected with a high dose (5 × 10^5^) of parasitized erythrocytes, one with each of the Mo7 and T2Bo [22] strains. They were then monitored as above on a daily basis for the first fifty dpi and on a biweekly basis thereafter until 10 months post-inoculation. The protocol for infection and animal handling used in this study [2, 4] was approved by the Institutional Animal Care and Use Committee (IACUC) at Washington State University.

### Immunofluorescence antibody assay

The IFA was performed as previously described [12, 23] using 50 μl of a 1/50 dilution of serum in serum dilution buffer (VMRD Inc., Pullman, WA, USA) and substrate slides prepared using red blood cells parasitized by two *B. bovis* strains, Mo7 and T2Bo. A positive result was defined as fluorescence equal to (1+) or greater (2 to 3+) than that of a weak positive control sample defined by IFA, western blot and RAP-1 cELISA after collection from a calf experimentally infected with Mo7 stain. A negative result was defined as comparable to the background fluorescence of a negative control serum collected from a *B. bovis*-negative herd in the northwestern US.

### SBP4 MI-ELISA

The rGST-SBP4-based MI-ELISA was prepared as follows. Fifty μL of diluted rGST-SBP4 fusion protein that gave approximately 0.08 optical density (OD) at 450 nm (A450) when tested against the negative reference serum were added to 96-well immunoassay plates (Costar, Vernon Hills, IL, USA). Plates were treated with 0.08 μg/well of glutathione-bovine serum albumin, incubated overnight at 4 °C, and then incubated for 2 h at 37 °C with 200 μl/well of blocking buffer (VMRD Inc.). The blocking buffer was subsequently removed, 50 μl per well of rGST-SBP4 diluted in phosphate buffered saline (PBS) containing a final concentration of 1% Triton X-100 (Sigma-Aldrich) was added to each well and incubated overnight at 4 °C. The plate was washed with PBS containing a final concentration of 0.05% Tween 20, incubated with blocking buffer for 3 h at 37 °C, and dried overnight. Finally, the antigen-coated plates were stored individually in polyester film bags (IMPAK Co. Los Angeles, CA, USA) at 4 °C until use.

For the SBP4 MI-ELISA testing procedure, 50 μl of undiluted test serum were added per well and incubated at room temperature for 30 min. Wells were then washed three times with 250 μl of wash buffer (VMRD Inc.) per well. Fifty microliters of HRP-conjugated rGST-SBP4 diluted in conjugate diluting buffer (VMRD Inc.) were added to each well of the plate, and the plates were incubated at room temperature for 30 min. After wells were again washed three times with 250 μl of wash buffer per well, 50 μl of tetramethylbenzidine substrate (SurModics, Eden Prairie, MN, USA) were added to each well, and the plates were incubated at room temperature for 15 min. The reactions were then stopped with 50 μl of 1.5% sodium fluoride solution (SurModics, Eden Prairie, MN, USA) per well. The optical densities (OD) of the assay wells were read at 450 nm (OD_450_) with a microplate spectrophotometer and results were calculated as the ratio of sample OD to negative control OD (S/N). Specifically, the OD_450_ of the sample in question was divided by the OD_450_ of a negative control analyzed in the same assay run.

### Western blot analysis for detection of bovine serum antibody to RAP-1

Western blot analysis to detect *B. bovis*-specific antibodies to RAP-1 in bovine sera was performed using recombinant RAP-1 (rRAP-1), according to the previously described method [13] with some modifications. Briefly, rRAP-1 was boiled for 3 min in sample buffer (DGel Sciences, Montreal, Canada) and separated by electrophoresis using sodium dodecyl sulfate-polyacrylamide gel (Bio-Rad, Hercules, CA, USA). Transfer to nitrocellulose was performed using standard techniques [24] and membranes were blocked in tris-tween-20 buffer containing 5% skim milk. Bovine serum antibody bound to the rRAP-1 band was detected with HRP-conjugated goat anti-bovine IgG (KPL, Gaithersburg, MD, USA) [15, 17].

### Data analysis

The diagnostic specificities of the SBP4 MI-ELISA and the RAP-1 cELISA were calculated as the percentage of sera IFA-negative for *B. bovis* that were also negative by the assay in question. Diagnostic sensitivity was the percentage of the IFA-defined *B. bovis*-positive sera having a positive result in the assay being evaluated. Receiver operating characteristic (ROC) curve and scatter plot analysis were performed using spreadsheet software (Excel software, Microsoft, Seattle, WA, USA) and R software from the R Foundation for Statistical Computing (http://www.r-project.org/) to evaluate the cutoff for positive and negative detection by the newly developed SBP4 MI-ELISA through comparison with the IFA-positive and -negative serum reference panels described above [25–27].

A binomial test [28] was used to determine if there were significant (*P <* 0.05) differences in sensitivity and specificity between the SBP4 MI-ELISA and RAP-1 cELISA or IFA test when testing various sets of serum samples. All statistical analyses were performed using the R software.

## Results

### Optimization of the SBP4 MI-ELISA and determination of the cutoff using ROC curve and scatter plot analysis

The SBP4 MI-ELISA was optimized by evaluation of several format variables including the antigen coating procedure, serum incubation time, wash buffer, and concentration of HRP/GST-SBP4 conjugate used to detect antibody binding. Optimization utilized a small set of six samples that included two known negative sera and four two-fold dilutions of a *B. bovis*-positive serum in negative serum ranging above and below the end-point of detection (Table 1). The optimized format included the use of 50 μl undiluted serum to maximize assay simplicity.

**Table 1:** Optimum calibration of the SBP4 MI-ELISA using a reference serum panel

Sample, dilution^a^	Mean OD	S/N ratio	Result^b^
Positive serum C167, 1/64	0.679	7.5	+
Positive serum C167, 1/128	0.394	4.3	+
Positive serum C167, 1/256	0.278	3.1	+
Positive serum C167, 1/512	0.187	2.1	–
Negative serum #37	0.123	1.3	–
Negative serum #312	0.149	1.6	–

*Abbreviations: OD* optical density, S *OD* of test sample, N *OD* of negative control

^a^Positive serum was diluted with negative serum

^b^Sample considered positive if the S/N ratio was ≥ 3

**Fig. 1:**
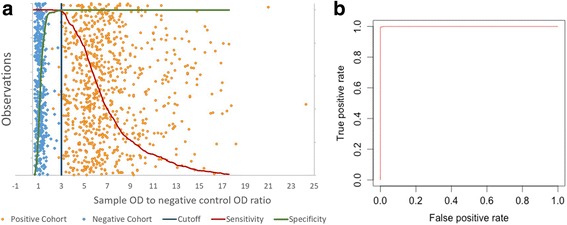
ROC curve analysis to determine optimal S/N ratio cutoff for the SBP4-based MI-ELISA. Data points in **a** represent 1,253 field sera categorized as *B. bovis* positive (*light blue*) or negative (*orange*) by IFA. *Dark blue* vertical line is 3 S/N ratio cutoff for the MI-ELISA. **b** is the analysis on the area on the ROC curve with 3 S/N ratio cutoff

Using this optimized SBP4 MI-ELISA format, S/N ratios were determined for 1,253 sera that were already categorized as either *B. bovis*-IFA positive or -IFA negative. Scatter plot and ROC curve analysis were carried out using the S/N ratio data and IFA categorization (Fig. 1). This analysis resulted in a maximum value for the combined sensitivity and specificity at a cutoff of approximately 2.0 (99.6% sensitivity, 98.4% specificity). The least difference between sensitivity and specificity occurred at a cutoff of approximately 2.4 (99.0% sensitivity, 98.6% specificity). The minimum cutoff that gave 100% sensitivity was 1.5, but this was at the expense of specificity dropping to 83.6%. The maximum cutoff that gave 100% specificity was 2.9 which resulted in 97.6% sensitivity. Using a more convenient cutoff of ≥ 3.0 yielded sensitivity and specificity values essentially identical to a cutoff of ≥ 2.9. The area under the ROC curve (AUC) with this cutoff was 0.9987, which is close to the perfect classification value of 1.00, indicating high accuracy of the 3.0 S/N ratio cutoff for classifying serum samples into positive or negative. Thus, a SBP4 MI-ELISA cutoff of ≥ 3 was selected for further use to provide maximum sensitivity with very good specificity (Fig. 1).

### Diagnostic specificity of the SBP4 MI-ELISA and the RAP-1 cELISA

To compare the specificity of the SBP4 MI-ELISA and the RAP-1 cELISA, 302 sera from northwestern U.S. herds maintained in barns free of *B. bovis* tick vectors and negative by IFA using two different *B. bovis* strains as IFA substrate were tested. All 302 sera were negative using both ELISA assays, generating no false positives and a resultant diagnostic specificity of 100% for both (Table 2). Furthermore, the SBP4 MI-ELISA also had 100% diagnostic specificity when evaluated with 32 IFA-negative sera collected from *B. bovis*-endemic regions of Mexico (Table 2). However, the RAP-1 cELISA had a lower diagnostic specificity of 93.8% as two of the same 32 IFA-negative sera were positive (Table 2).

**Table 2:** Diagnostic specificities of the SBP4 MI-ELISA and RAP-1 cELISA for *B. bovis*. All sera were negative based on immunofluorescent antibody assay

Sample origin (number)	SBP4 MI-ELISA^a^	RAP-1 cELISA^b^
Northwestern United States (302)	100% (302/302)	100% (302/302)
Mexico (*B. bovis* endemic regions) (32)	100% (32/32)	93.8% (30/32)
Texas (93)	100% (93/93)	90.3% (84/93)

^a^Positive cutoff ≥ 3 S/N ratio

^b^Positive cutoff ≥ 21% inhibition

Ninety-three of 94 additional sera collected from Texas herds as potential negative samples were negative by IFA, with the remaining sample being weakly IFA positive. Nine of the 93 IFA negative sera were positive by RAP-1 cELISA (90.3% specificity) while none were positive by SBP4 MI-ELISA (100% specificity). All nine IFA-negative sera with discrepant results between the RAP-1 cELISA and the SBP4 MI-ELISA were negative in RAP-1 western blot analysis (Additional file 1: Figure S1), supporting the possibility of false positive RAP-1 cELISA results with these nine sera. However, it is prudent to admit that western blot is not a validated diagnostic method for *B. bovis* infection, but only an additional assay used to support the results in MI-ELISA. Interestingly, the one weak IFA positive serum from the 94 Texas samples was negative by RAP-1 cELISA and SBP4 MI-ELISA, introducing the possibility that this serum was IFA false positive, although this was not proven. Ultimately, the SBP4 MI-ELISA had significantly (*P*-value = 0.006) better diagnostic specificity than the RAP-1 cELISA against sera from Texas using IFA as the reference assay.

### Diagnostic sensitivity of the SBP4 MI-ELISA and the RAP-1 cELISA

To evaluate the sensitivity of the SBP4 MI-ELISA and the RAP-1 cELISA against well-characterized positive sera, 402 IFA-positive sera collected at various DPIs from six cattle experimentally infected with one of three different *B. bovis* strains were analyzed. Of the 402 sera, 335 sera were strong IFA positives while the remaining 67 sera were weak IFA positives. The RAP-1 cELISA had a sensitivity of 84.6%, with 340 of the 402 IFA positive sera being RAP-1 cELISA positive (Table 3). The SBP4 MI-ELISA had a sensitivity of 100% as all 402 IFA positive sera were positive. For these experimentally infected calves, the SBP4 MI-ELISA had significantly (*P* < 0.001) better sensitivity than the RAP-1 cELISA.

**Table 3:** Diagnostic sensitivities of the SBP4 MI-ELISA and RAP-1 cELISA for *B. bovis*. All sera were positive based on immunofluorescent antibody assay. The cutoff for a SBP4 MI-ELISA positive result was a ≥ 3 S/N ratio, the cutoff for RAP-1 cELISA was ≥ 21% inhibition, and the cutoff for IFA was ≥ 1+ fluorescence

Sample origin (number)	SBP4 MI-ELISA	RAP-1 cELISA
Sera collected from six experimentally-infected calves (402)	100% (402/402)	84.6% (340/402)
Sera collected from herds vaccinated and/or challenged with virulent W strain in Australia (228)	95.2% (217/228)	86.0% (196/228)
Sera collected from *B. bovis*-endemic regions of Mexico (598)	98.7% (590/598)	60.0% (359/598)

One hundred seventy-seven of 228 sera collected from cattle herds in Australia that were vaccinated with an attenuated *B. bovis* strain or challenged with the pathogenic W strain were strong positive by IFA, and the remaining 51 sera were weak positive. Analyses with all of these 228 sera resulted in 95.2% and 86.0% diagnostic sensitivity for the SBP4 MI-ELISA and the RAP-1 cELISA, respectively (Table 3). Thus, the SBP4 MI-ELISA had significantly (*P* ≤ 0.004) better diagnostic sensitivity than the RAP-1 cELISA against these sera.

Three hundred eighty-eight of 598 IFA positive sera collected from endemic regions in Mexico were strongly IFA-positive and the rest were weak IFA-positive. The diagnostic sensitivity of the SBP4 MI-ELISA was 98.7% while diagnostic sensitivity of the RAP-1 cELISA was 60.0% for all 598 IFA-positive sera (Table 3). The SBP4 MI-ELISA had significantly (*P* < 0.001) better diagnostic sensitivity than the RAP-1 cELISA for these sera.

### Analytical specificity of the SBP4 MI-ELISA against sera positive for *Babesia bigemina* and *Anaplasma marginale*

The analytical specificity of the SBP4 MI-ELISA was evaluated for sera positive to *B. bigemina* (*n* = 25) and *Anaplasma marginale* (*n* = 25)*,* which are other tick-borne intraerythrocytic parasites commonly co-infecting with *B. bovis* in cattle from endemic areas. All of these sera were negative in the SBP4 MI-ELISA, demonstrating suitable analytical specificity against closely related and/or frequently co-infecting intra-erythrocyte parasites (Additional files 2 and 3: Tables S1, S2).

### Long-term monitoring of parasitemia and antibody responses in calves infected with a high dose of *Babesia bovis* T2Bo or Mo7 strain

**Fig. 2:**
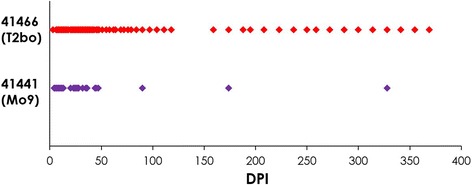
Long-term monitoring of *B. bovis* parasitemia in calves challenged with a high dose of the T2Bo or Mo7 strain. The red dots depict positive parasitemia days of calf 41466 (infected with the T2Bo strain of *B. bovis)* and purple dots the positive parasitemia days of calf 41441 (infected with Mo9). Parasitemia was monitored by nested PCR on a daily basis for the first fifty DPI and on a biweekly basis thereafter until 10 months pi. *Abbreviation*: DPI, days post-infection

Blood samples from two calves experimentally infected with a high dose of *B. bovis* (5 × 10^5^ infected erythrocytes; one with Mo7 and one with the T2Bo strain) were evaluated daily for the first 33 days post-infection and then biweekly until 12 months post-inoculation by nested PCR (Fig. 2). Initial parasitemia detection by nested PCR in these two calves was four DPI (Fig. 2). Calf C41466, infected with the T2Bo strain, had more frequent positive nested PCR results than calf C41441, infected with the cloned moderately attenuated Mo7 strain. After the initial positive nested PCR at four DPI, there were 61 (81.3%) versus 25 (33.3%) PCR positive time points out of 75 tested for C41466 and C41441, respectively (Fig. 2).

**Fig. 3:**
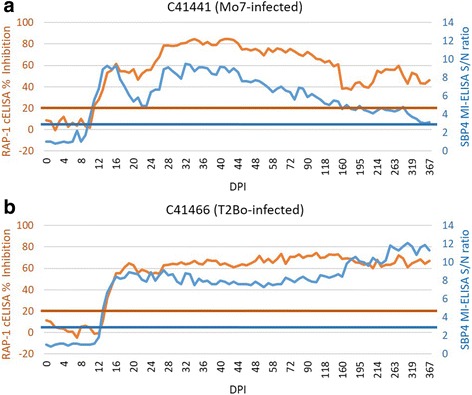
Long-term monitoring of *B. bovis* antibody responses by the SBP4-based MI-ELISA and the RAP-1-based cELISA in calves challenged with a high dose of the Mo7 (**a**. C41441) or T2Bo (**b**. C41466) strain. S/N ratio indicates sample optical density to negative control OD ratio for the SBP4 MI-ELISA, and %I indicates % inhibition for the RAP-1 cELISA. Blue and red horizontal lines are 3 S/N ratio cutoff for the MI-ELISA or 21% inhibition cutoff for the RAP-1 cELISA, respectively. *Abbreviations*: DPI, days post-infection; OD, optical density

**Fig. 4:**
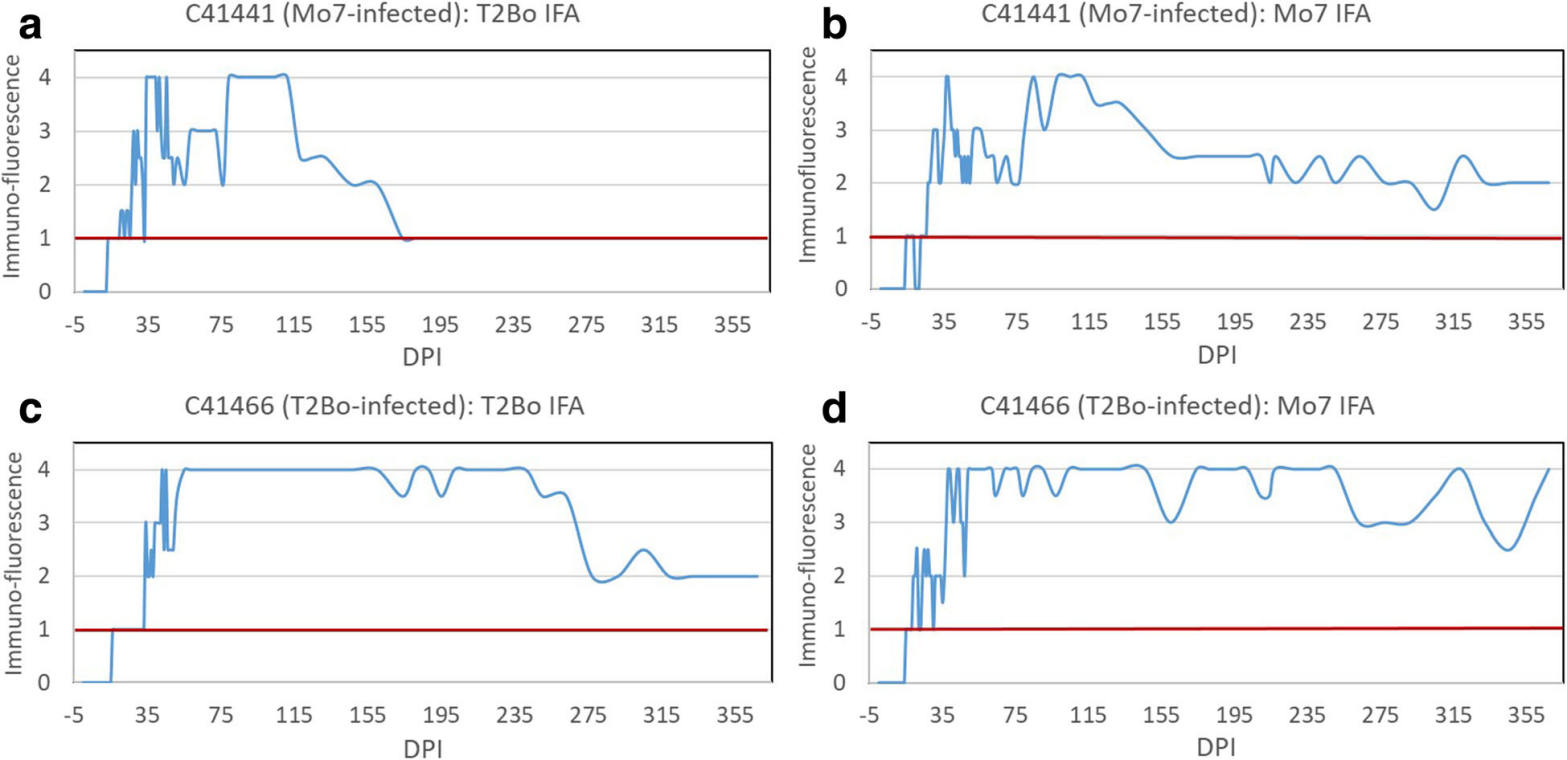
Long-term monitoring of *Babesia bovis* antibody responses by IFA in calves challenged with a high dose of the T2Bo or Mo7 strain. Calf C41441 time point sera were analyzed on IFA slides made with **a.** T2Bo and **b**. Mo7 strains. Calf C41466 time point sera were analyzed on IFA slides made with **c**. T2Bo and **d**. Mo7 strains. A positive IFA result was defined as fluorescence ≥ 1+. *Abbreviation*: DPI, days post-infection

The timing of initial antibody detection in these experimentally infected calves was slightly variable depending on the assay and the *B. bovis* strain used for infection. The calf infected with high dose Mo7 had detectable antibody beginning at 10, 11 and 14 DPI by SBP4 MI-ELISA (Fig. 3a), RAP-1 cELISA (Fig. 3a) and IFA (Fig. 4a, b), respectively. The calf infected with high dose T2Bo had detectable antibody beginning at 13, 14 and 15 DPI by the same three assays, respectively (Figs. 3b and 4c, d). Thus, the difference between the SBP4 MI-ELISA and the RAP-1 cELISA in the timing of initial antibody detection was only one day for both strains.

*Babesia bovis*-specific antibody responses after initial positive detection were maintained for longer than 12 months post-infection according to all three assays (Figs. 3 and 4). The long-term pattern of antibody response in the T2Bo-infected calf C41466 was consistently robust and increased for 12 months with only minor fluctuations as assessed by the SBP4 MI-ELISA (Fig. 3b). Positive antibody responses were observed at all of 66, 65 and 65 time points tested after the first positive detection by SBP4 MI-ELISA, RAP-1 cELISA, and IFA, respectively (Figs. 3 and 4). Using sera collected from C41466 after the first positive result by SBP4 MI-ELISA, the percentage positive by nested PCR, SBP4 MI-ELISA, RAP-1 cELISA and IFA were 87.9%, 100%, 98.5% and 97.0%, respectively. The less than 100% by RAP-1 cELISA and IFA were due to some negative tests just after the SBP4 MI-ELISA became positive.

Although consistently positive following seroconversion, the antibody response detected by the SBP4 MI-ELISA in calf C41441 (challenged with the attenuated Mo7 strain) was less robust and gradually decreased to near the S/N ratio cutoff, but remained positive, at 12 months post-infection (Fig. 3a). A similar pattern was observed using the RAP-1 cELISA (Fig. 3a) and IFA (Fig. 4a, b). C41441 had positive responses at all of 69, 68 and 66 time points tested after the first positive detection by SBP4 MI-ELISA, RAP-1 cELISA, and IFA, respectively (Figs. 3a, 4a, b). Using time points collected in C41441 after the first positive result in SBP4 MI-ELISA, the percentage positive by nested PCR, SBP4 MI-ELISA, RAP-1 cELISA, and IFA were 27.5%, 100%, 98.6% and 94.2%, respectively. The less than 100% by RAP-1 cELISA and IFA were due to some negative tests just after the SBP4 MI-ELISA became positive. Since the two calves were experimentally infected and only intermittently positive by PCR, these results demonstrated that the three antibody assays were far more effective than the nested PCR in determining infection status, especially for calf C41441.

### Long-term monitoring of parasitemia and antibody responses in calves infected with a low dose of *Babesia bovis* Tf-137-4 or Mo7 strain

**Fig. 5:**
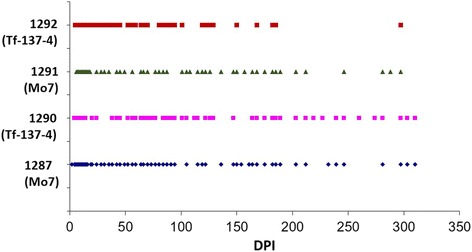
Long-term monitoring of *B. bovis* parasitemia in calves challenged with a low dose of the Tf-137-4 or Mo7 strain. The red and pink dots depicting positive parasitemia days of calves C1292 and C1290 (infected with the Tf-137-4 strain of *B. bovis*), respectively and green and blue dots the positive parasitemia days of calves C1291 and C1287 (infected with Mo9), respectively. Parasitemia was monitored by nested PCR on a daily basis for the first twenty DPI and on a biweekly basis thereafter until 11 months post-infection. *Abbreviation*: DPI, days post-infection

**Fig. 6:**
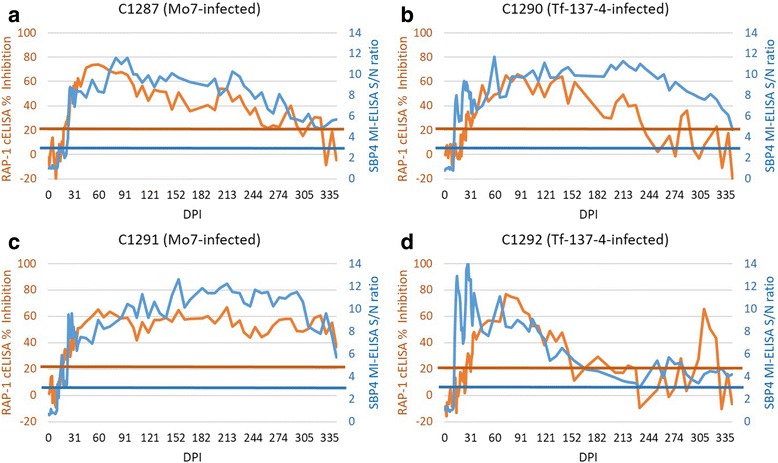
Long-term monitoring of *B. bovis* antibody responses by the SBP4-based MI-ELISA and the RAP-1-based cELISA in calves challenged with a low dose of the Mo7 (**a**. C1287, **c**. C1291) or Tf-137-4 (**b**. C1290, **d**. C1292) strain. S/N ratio indicates sample optical density to negative control OD ratio for the SBP4 MI-ELISA. Blue horizontal line is 3 S/N ratio cutoff for the MI-ELISA. %I indicates % inhibition for the RAP-1 cELISA. Red horizontal line is 21% inhibition cutoff for the RAP-1 cELISA. *Abbreviations*: DPI, days post-infection; OD, optical density

**Fig. 7:**
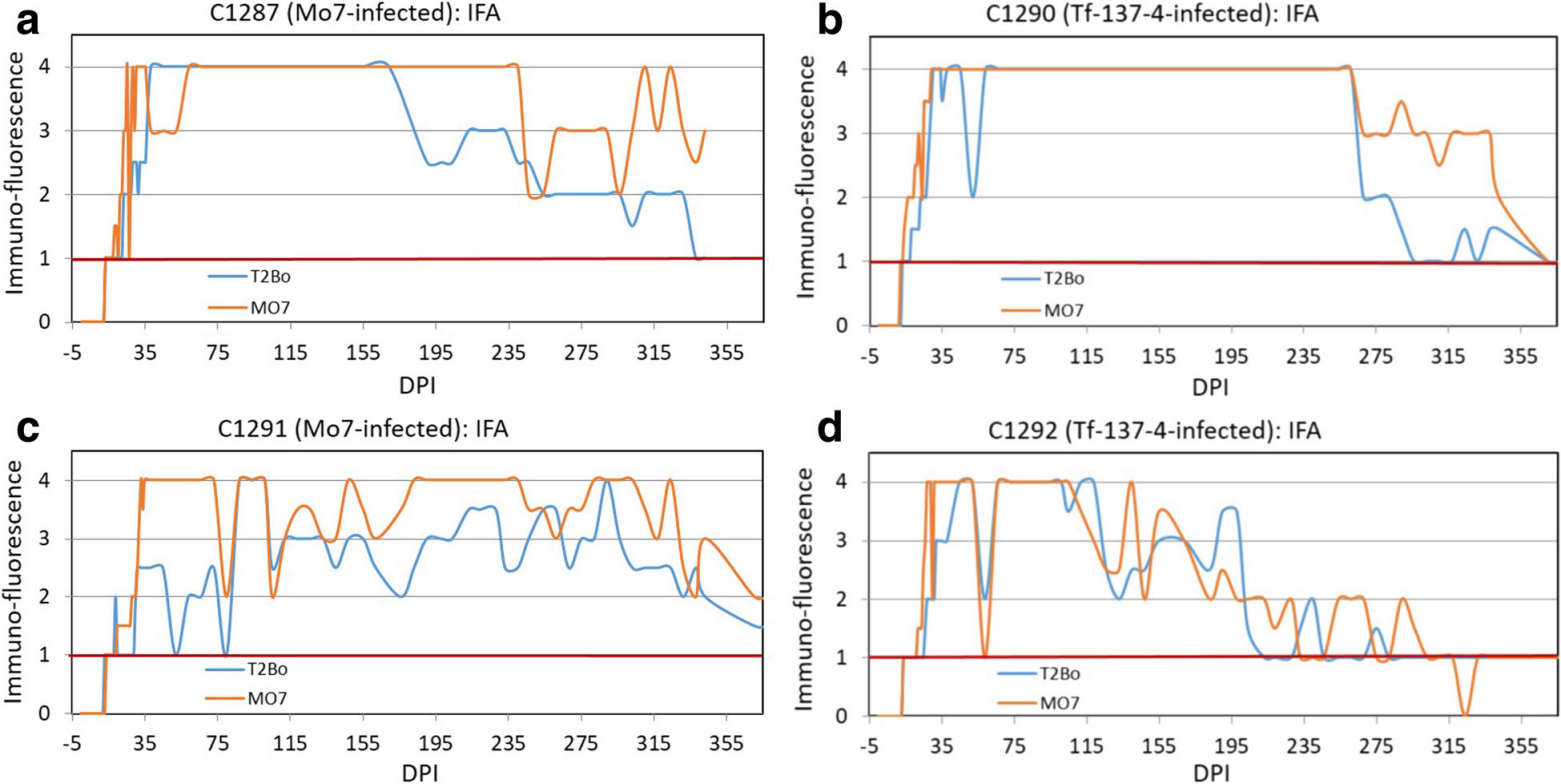
Long-term monitoring of *B. bovis* antibody responses by IFA in calves challenged with a low dose of the Mo7 (**a**. C1287, **c**. C1291) or Tf-137-4 (**b**. C1290, **d**. C1292) strain. A positive IFA result was defined as fluorescence ≥ 1+. *Abbreviation*: DPI, days post-infection

Four calves experimentally infected with a low dose (5 × 10^3^ infected erythrocytes) of *B. bovis*, two with the Mo7 strain (C1287 and C1291) and two with the attenuated Tf-137-4 strain (C1290 and C1292), had variable parasitemia for longer than 11 months when tested by nested PCR, demonstrating long-term persistence of *B. bovis* after infection (Fig. 5). Initial parasitemia detection by nested PCR in these four calves was 2 to 6 DPI (Fig. 5). The timing of initial antibody detection in low dose *B. bovis*-infected calves was notably variable, being both assay- and strain-dependent (Figs. 6 and 7). The two calves infected with Tf-137-4 had detectable *B. bovis*-specific antibodies at 12 and 14–15 DPI when tested by SBP4 MI-ELISA (Fig. 6b, d) and IFA (Fig. 7b, d), respectively. However, the first positive antibody detection by the RAP-1 cELISA in the same calves was at 26 DPI, representing an 11 to 14 day delay in detection in calves infected with a low dose of an attenuated strain (Fig. 6b, d). The two Mo7-infected calves had detectable antibody at 13 and 13–14 DPI when tested by the SBP4 MI-ELISA (Fig. 6a, c) and IFA (Fig. 7a, c), respectively. However, the earliest positive antibody detection in the same calves was at 18 DPI using the RAP-1 cELISA (Fig. 6a, c), a four to five day delay in detection.

Following initial detection in these four calves, the long-term pattern of parasitemia determined by nested PCR was intermittent. After the first positive SBP4 MI-ELISA result at 13 DPI, the Mo7-infected calves had 84.7% (50 of 59 time points) and 69.5% (41 of 59) nested PCR-positive time points (Fig. 5). Using the same time points, 100% were SBP4 MI-ELISA positive (Fig. 6), 83.1 to 86.4% RAP-1 cELISA positive (Fig. 6), and 98.3 to 100% positive with IFA (Fig. 7). Tf-137-4-infected calves had 78.3% (47 of 60 time points) and 56.7% (34 of 60) nested PCR-positive time points after 12 DPI, the first positive SBP4 MI-ELISA result (Fig. 5). Using the same time points, 100%, 50.0 to 55.0% and 95.0 to 96.7% were antibody positive by SBP4 MI-ELISA (Fig. 6), RAP-1 cELISA (Fig. 6) and IFA (Fig. 7), respectively. Positive antibody responses after initial detection in all four calves were maintained at all time points up to 12 months when tested by the SBP4 MI-ELISA (Fig. 6). Following a one to three day delay in antibody detection as compared to SBP4 MI-ELISA, IFA also had persistent positive detection at all time points (Fig. 7). However, antibody detected by the RAP-1 cELISA in three of the same four calves dropped rapidly below the cutoff by 155 DPI in C1292, 240 in C1290 and 303 in C1287 (Fig. 6a, b, d), suggesting insufficient reliability of the RAP-1 cELISA for monitoring the inapparent carrier stage of *B. bovis* infection.

## Discussion

In the present study, an MI-ELISA was developed using recombinant SBP4 as the antigen for coating as well as for detection when conjugated with HRP. The SBP4 MI-ELISA had much better diagnostic performance in this study compared to the RAP-1 cELISA [6, 12] as well as in comparison to that reported for the indirect ELISA based on SBP4 [15]. The cutoff for positive or negative antibody detection used in this assay was 3.0 S/N ratio which gave an excellent combination of 100% specificity and 97.6% sensitivity when tested with 1,253 diverse sera collected from endemic, epidemic and non-endemic areas. In addition, the range of acceptable S/N ratio cutoff giving excellent (≥97%) prediction in both specificity and sensitivity was wide (between 1.8 and 3.4) demonstrating good resolution of the assay in differentiating positives from negatives. The SBP4 MI-ELISA had 37% higher diagnostic sensitivity than the RAP-1 cELISA when tested with sera collected from *B. bovis*-endemic regions in Mexico. It also had better diagnostic specificity than the RAP-1 cELISA when tested with sera from the southern U.S. (100 vs 90.3%) and Mexico (100 vs 93.8%), although diagnostic specificity was 100% for both when tested with sera from non-endemic northwestern U.S. herds kept in tick-free barns.

Most cattle survive acute *B. bovis* infection, and some studies have demonstrated that recovered cattle become clinically inapparent carriers that could serve as reservoirs for intra- and inter-herd transmission [1, 5, 6, 9]. Unlike *B. bigemina*, *B. bovis* is able to escape the immune system of the host using rapid antigenic variation, cytoadhesion and capillary sequestration, and binding of host proteins to the surface of infected red blood cells [29]. It is believed that these mechanisms contribute to the establishment of persistent infections and can result in the development of long-term, fluctuating immune responses. If persistent infections are characterized by very low levels of circulating antibodies and often undetectable parasitemia, then detection may require exquisitely sensitive methods. To this end, an important objective in this study was to evaluate how the SBP4 MI-ELISA, RAP-1 cELISA, and IFA perform against samples collected from calves with fluctuating kinetics of parasitemia. This included calves experimentally infected with high and low doses of three different *B. bovis* strains, both attenuated and pathogenic, over the duration of 11 to 12 months. All six experimentally infected animals had recurrent parasitemia lasting more than ten months post-infection, including those infected with a low dose of attenuated *B. bovis* strains. This indicates that the capacity to cause persistent infection does not depend on pathogenicity or challenge dose, although the five animals infected with attenuated strains had no clinical signs or thrombocytopenia after the first acute parasitemia.

More frequent parasitemia was detected by PCR in the calf challenged with the pathogenic strain T2Bo relative to calves challenged with the attenuated strains Mo7 and Tf-137-4. This suggests that parasitemia detection-based diagnostics such as PCR lack reliability, likely due to fluctuation of parasitemia from sequestration and antigenic variation [29]. However, all six calves maintained *B. bovis* antibody responses throughout the entire 11 to 12 month monitoring period when analyzed by the SBP4 MI-ELISA and IFA. Persistence of antibody responses specific for *B. bovis* SBP4 even at DPIs with no detectable parasitemia by nested PCR clearly indicates the relative advantage of these antibody-based diagnostics over the parasitemia detection used. Extensive use of antibody detection depends on the availability of an accurate, high-throughput serology assay with more objective and less isolate-dependent interpretation than IFA, such as the SBP4 MI-ELISA described in this study. The diagnostic sensitivity and specificity of the MI-ELISA in this study were much higher than the performance (84.5% sensitivity and 86.2% specificity) reported in a previous study [15] using a similar rSBP4 design in a conventional indirect ELISA format. This contrast could be related to 1) a higher quality steric presentation of poly epitopes using glutathione-BSA catcher for solid phase coating of rGST-SBP4, and 2) higher binding efficiency of *B. bovis*-specific detection conjugate in liquid phase. In the MI-ELISA, the wells of the immunoassay plate are coated with glutathione-BSA, followed by the rGST-SBP4 fusion protein resulting in oriented steric presentation of poly SBP4 epitopes as compared to the direct random coating of rGST-SBP4 on microplate wells in the conventional indirect ELISA. In addition, the MI-ELISA uses a HRP-conjugated rGST-SBP4 fusion protein as the detecting reagent, thus the poly-epitope binding results in increased specificity as compared to the conventional indirect ELISA. Side by side comparison of two assay formats was not included in this manuscript, but technical and performance features of the MI-ELISA versus conventional indirect ELISA need to be further evaluated in a systematic comparative study. This assay will be much more reliable tool for identifying animals in the carrier stage of *B. bovis* infection than PCR-based parasitemia detection or antibody detection by the RAP-1 cELISA.

## Conclusions

The results of this study demonstrate that the SBP4 MI-ELISA has very high diagnostic specificity and sensitivity when evaluated with diverse sera collected from bovine herds in non-endemic, endemic, and epidemic areas. This SBP4 MI-ELISA can consistently detect *B. bovis*-specific antibodies in calves with acute as well as long-term carrier infections, which makes it a pivotal tool for identifying *B. bovis*-infected cattle in endemic areas and preventing their movement to non-endemic and free areas.

## Additional files

Additional file 1: Figure S1. Cattle sera positive by the RAP-1-based cELISA but negative by the SBP4-based MI-ELISA and IFA had negative results by Western blot analysis, suggesting possible false positive results in the cELISA. A. Molecular weight marker (48 to 180 Kd), B. K42-#21, C. W31-#Y-3, D. W31-#Y-11, E. W31-#0-3, F. W31-#Y-9, G. W31-#0-9, H. W31-#Y-10, I. W31-#Y-15, J. P21-#224, K. positive control serum with a band at 75kd representing *B. bovis* RAP-1 protein, J. negative control serum. **Figure S2.** Technical difference between the modified indirect ELISA and conventional indirect ELISA using rGST-SBP4 was illustrated in this figure. (DOCX 645 kb)
Additional file 2: Table S1. Excellent analytical specificity of the SBP4 MI-ELISA was demonstrated by no cross-reactivity against *Babesia bigemina* antibody-positive sera. *Babesia bigemina* antibody-positive sera collected from calves experimentally infected with Puerto Rico strain were characterized by IFA before use. (DOC 63 kb)
Additional file 3: Table S2. Excellent analytical specificity of the SBP4 MI-ELISA was demonstrated by no cross-reactivity against *Anaplasma marginale* antibody-positive sera. *Anaplasma marginale* antibody-positive sera collected from calves experimentally infected with St. Mary strain were characterized by a commercial cELISA before use. (DOC 64 kb)

## Abbreviations

AUC: Area under the receiver operating characteristic curve
cELISA: Competitive blocking enzyme-linked immunosorbent assay
CF: Complement fixation
DPI: Days post-infection
GST: Gluththione S-transferase
HRP: Horseradish peroxidase
IFA: Indirect immunofluorescence assay
IPTG: Isopropyl-β-D-thiogalactopyranoside
MI-ELISA: Modified indirect enzyme-linked immunosorbent assay
MLV: Modified live vaccine
OD: Optical density
PCV: Packed cell volume
RAP-1: Rhoptry-associated protein 1
ROC: Receiver operating characteristic
S/N: The ratio of sample OD to negative control OD
SBP4: Spherical body protein 4
