# Supplementary material for: A novel modified-indirect ELISA based on spherical body protein 4 for detecting antibody during acute and long-term infections with diverse Babesia bovis strains
Source: Parasit Vectors. 2017 Feb 13;10:77. doi: 10.1186/s13071-017-2016-9 (PMC5307855; doi:10.1186/s13071-017-2016-9)

**Additional file 1. Figure S1.** Cattle sera positive by the RAP-1-based cELISA but negative by the SBP4-based MI-ELISA and IFA had negative results by Western blot analysis, suggesting possible false positive results in the cELISA.

A. Molecular weight marker (48 to 180 Kd), B. K42-#21, C. W31-#Y-3, D. W31- #Y-11, E. W31-#0-3, F. W31- #Y-9, G. W31-#0-9, H. W31- #Y-10, I. W31-#Y-15, J. P21-#224, K. positive control serum with a band at 75kd representing *B. bovis* RAP-1 protein, J. negative control serum


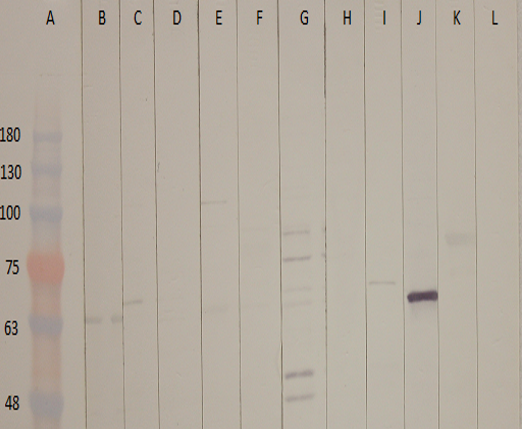


**Additional file 1. Figure S2.** Technical difference between the modified indirect ELISA and conventional indirect ELISA using rGST-SBP4 was illustrated in this figure.


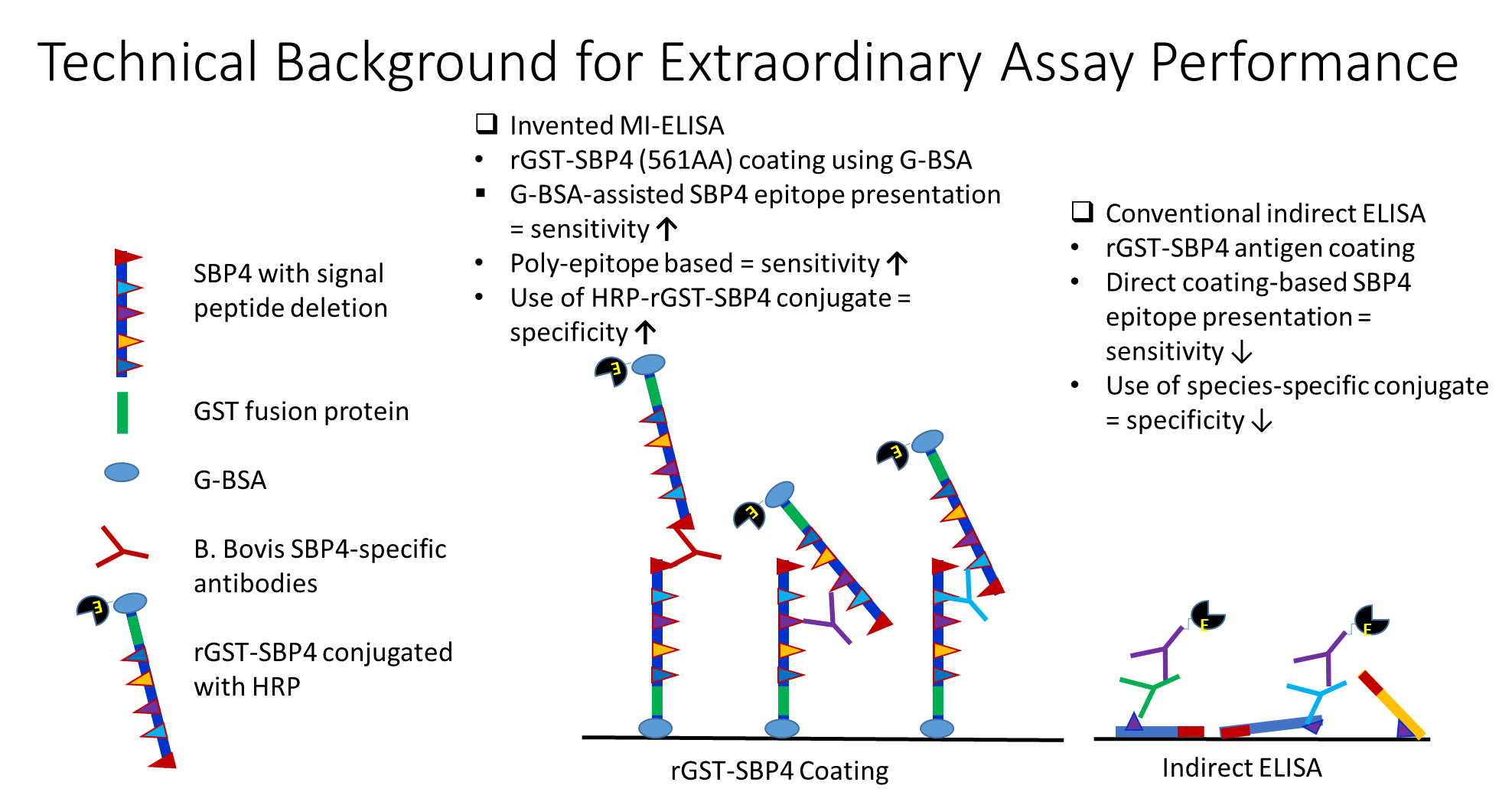

Supplement: Additional file 1: Figure S1. — Cattle sera positive by the RAP-1-based cELISA but negative by the SBP4-based MI-ELISA and IFA had negative results by Western blot analysis, suggesting possible false positive results in the cELISA. A. Molecular weight marker (48 to 180 Kd), B. K42-#21, C. W31-#Y-3, D. W31-#Y-11, E. W31-#0-3, F. W31-#Y-9, G. W31-#0-9, H. W31-#Y-10, I. W31-#Y-15, J. P21-#224, K. positive control serum with a band at 75kd representing B. bovis RAP-1 protein, J. negative control serum. Figure S2. Technical difference between the modified indirect ELISA and conventional indirect ELISA using rGST-SBP4 was illustrated in this figure. (DOCX 645 kb) [file 13071_2017_2016_MOESM1_ESM.docx]
