# Supplementary material for: A novel modified-indirect ELISA based on spherical body protein 4 for detecting antibody during acute and long-term infections with diverse Babesia bovis strains
Source: Parasit Vectors. 2017 Feb 13;10:77. doi: 10.1186/s13071-017-2016-9 (PMC5307855; doi:10.1186/s13071-017-2016-9)
Supplement: Additional file 2: Table S1. — Excellent analytical specificity of the SBP4 MI-ELISA was demonstrated by no cross-reactivity against Babesia bigemina antibody-positive sera. Babesia bigemina antibody-positive sera collected from calves experimentally infected with Puerto Rico strain were characterized by IFA before use. (DOC 63 kb) [file 13071_2017_2016_MOESM2_ESM.doc]

**Additional file 1. Table S1.** Excellent analytical specificity of the SBP4 MI-ELISA was demonstrated by no cross-reactivity against *Babesia bigemina* antibody-positive sera. *Babesia bigemina* antibody-positive sera collected from calves experimentally infected with Puerto Rico strain were characterized by IFA before use.

|  | *B. bovis* SBP4 MI-ELISA* | | *B. bigemina* RAP-1 cELISA** | |
| --- | --- | --- | --- | --- |
| Sample ID | S/N ratio | Result | % inhibition | Result |
| 1 | 1.1 | - | 66.4 | + |
| 2 | 1.9 | - | 51.3 | + |
| 3 | 1.8 | - | 57.6 | + |
| 4 | 1.3 | - | 46.6 | + |
| 5 | 1.6 | - | 54.9 | + |
| 6 | 1.6 | - | 54.9 | + |
| 7 | 1.1 | - | 56.9 | + |
| 8 | 1.9 | - | 51.1 | + |
| 9 | 1.6 | - | 50.5 | + |
| 10 | 1.3 | - | 44.9 | + |
| 11 | 1.3 | - | 67.6 | + |
| 12 | 1.2 | - | 77.6 | + |
| 13 | 1.1 | - | 80.2 | + |
| 14 | 1.2 | - | 81.8 | + |
| 15 | 1.1 | - | 81.9 | + |
| 16 | 1.2 | - | 80.4 | + |
| 17 | 1.2 | - | 78.4 | + |
| 18 | 1.1 | - | 80.0 | + |
| 19 | 1.1 | - | 76.6 | + |
| 20 | 1.3 | - | 65.3 | + |
| 21 | 1.1 | - | 79.8 | + |
| 22 | 1.1 | - | 61.7 | + |
| 23 | 1.1 | - | 56.4 | + |
| 24 | 1.2 | - | 52.5 | + |
| 25 | 1.1 | - | 54.0 | + |
| *B. bovis* (+) | 7.6 | + | NA | NA |
| *B. bovis* (-) | 1.0 | - | NA | NA |
| *B. bigemina* (+) | NA | NA | 55.3 | + |
| *B. bigemina* (-) | NA | NA | 0.0 | - |

* Positive cut-off ≥3 S/N ratio

** Positive cut-off ≥21% inhibition.
