# Supplementary material for: A novel modified-indirect ELISA based on spherical body protein 4 for detecting antibody during acute and long-term infections with diverse Babesia bovis strains
Source: Parasit Vectors. 2017 Feb 13;10:77. doi: 10.1186/s13071-017-2016-9 (PMC5307855; doi:10.1186/s13071-017-2016-9)
Supplement: Additional file 3: Table S2. — Excellent analytical specificity of the SBP4 MI-ELISA was demonstrated by no cross-reactivity against Anaplasma marginale antibody-positive sera. Anaplasma marginale antibody-positive sera collected from calves experimentally infected with St. Mary strain were characterized by a commercial cELISA before use. (DOC 64 kb) [file 13071_2017_2016_MOESM3_ESM.doc]

**Additional file 3. Table S2.** Excellent analytical specificity of the SBP4 MI-ELISA was demonstrated by no cross-reactivity against against *Anaplasma marginale* antibody-positive sera. *Anaplasma marginale* antibody-positive sera collected from calves experimentally infected with St. Mary strain were characterized by a commercial cELISA before use.

|  | | *B. bovis* SBP4 MI-ELISA*** | | | *A. marginale* MSP5 cELISA**** | |
| --- | --- | --- | --- | --- | --- | --- |
| Sample ID | S/N ratio | | Result | % inhibition | | Result |
| 1 | 0.7 | | - | 93.4 | | + |
| 2 | 1.1 | | - | 91.7 | | + |
| 3 | 1.1 | | - | 93.6 | | + |
| 4 | 1.6 | | - | 48.2 | | + |
| 5 | 2.7 | | - | 74.9 | | + |
| 6 | 2.2 | | - | 80.3 | | + |
| 7 | 2.2 | | - | 83.1 | | + |
| 8 | 2.1 | | - | 82.7 | | + |
| 9 | 2.3 | | - | 85.6 | | + |
| 10 | 2.2 | | - | 85.2 | | + |
| 11 | 1.8 | | - | 89.1 | | + |
| 12 | 1.7 | | - | 87.2 | | + |
| 13 | 2.0 | | - | 88.2 | | + |
| 14 | 1.6 | | - | 88.1 | | + |
| 15 | 1.5 | | - | 86.9 | | + |
| 16 | 1.5 | | - | 87.1 | | + |
| 17 | 1.2 | | - | 94.5 | | + |
| 18 | 1.1 | | - | 89.0 | | + |
| 19 | 1.6 | | - | 91.0 | | + |
| 20 | 1.3 | | - | 93.0 | | + |
| 21 | 1.8 | | - | 90.8 | | + |
| 22 | 1.1 | | - | 87.4 | | + |
| 23 | 1.3 | | - | 91.9 | | + |
| 24 | 2.1 | | - | 84.5 | | + |
| 25 | 1.8 | | - | 81.9 | | + |
| *B. bovis* (+) | 4.4 | | + | NA | | NA |
| *B. bovis* (-) | 1 | | - | NA | | NA |
| *Anaplasma* (+) | NA | | NA | 81.7 | | + |
| *Anaplasma* (-) | NA | | NA | 0 | | - |

* Positive cut-off ≥3 S/N ratio

** Positive cut-off ≥30% inhibition
